# Uncertainty-induced instantaneous speed and acceleration of a levitated particle

**DOI:** 10.1038/s41598-021-97663-z

**Published:** 2021-09-14

**Authors:** Luca Ornigotti, Radim Filip

**Affiliations:** Department of Optics, Palacký University, 17. Listopadu 1192/12, 771 46 Olomouc, Czech Republic

**Keywords:** Optics and photonics, Condensed-matter physics, Optical physics, Statistical physics, thermodynamics and nonlinear dynamics

## Abstract

Levitating nanoparticles trapped in optical potentials at low pressure open the experimental investigation of nonlinear ballistic phenomena. With engineered non-linear potentials and fast optical detection, the observation of autonomous transient mechanical effects, such as instantaneous speed and acceleration stimulated purely by initial position uncertainty, are now achievable. By using parameters of current low pressure experiments, we simulate and analyse such uncertainty-induced particle ballistics in a cubic optical potential demonstrating their evolution, faster than their standard deviations, justifying the feasibility of the experimental verification. We predict, the maxima of instantaneous speed and acceleration distributions shift alongside the potential force, while the maximum of position distribution moves opposite to it. We report that cryogenic cooling is not necessary in order to observe the transient effects, while a low uncertainty in initial particle speed is required, via cooling or post-selection, to not mask the effects. These results stimulate the discussion for both attractive stochastic thermodynamics, and extension of recently explored quantum regime.

## Introduction

Stochastic levitating optomechanics in vacuum is a dynamically expanding experimental platform with a unique potential to test and exploit strong nonlinear motional effects without any friction, and bring them close to the quantum domain. This uniqueness arises from the possibility to combine manipulation of the optical trapping potential by a spatial light modulator, inducing new unexplored nonlinearities, and fast optical measurement to verify rapid transient effects using modern optical detectors. At low pressure, it allows direct observation of stochastic underdamped mechanical phenomena^1–7^, which allow access to the instantaneous particle speed not measurable in the overdamped motion^8,9^. In the transient ballistic regime, the surrounding environment does not modify the statistics of the instantaneous velocity^8,10,11^. Moreover, the initial uncertainty of the levitating particle can be controlled, by postselection^12^, feedback cooling^13–16^ and ultimately, by coherent scattering to the mechanical ground states^17–19^. All these key ingredients encourage broader investigation of the fundamental aspects of statistical mechanics^20,21^ and accelerate development of applications in mechanical sensing^5,22–24^ and thermodynamical engines^25,26^. Recently, the highly unstable motion of a levitating particle in the cubic potential has been analysed^27,28^ and experimentally verified^29,30^ in the overdamped regime. It was demonstrated that the mean particle position, induced by the initial position uncertainty, increases faster than that uncertainty. Atypically, the position distribution maximum shifts in the opposite direction to the mean. These investigations have already been stimulating experimentally verifiable thermodynamical consequences^27^.

In the low pressure limit the particle is deep in the underdamped regime so that the instantaneous particle speed and acceleration become new transient quantities to be first explored and later exploited for applications. In this paper we simulate and analyse nonlinear ballistic effects for instantaneous velocity and acceleration induced by the initial position uncertainty, and predict the experimental regime where such phenomena are visible using parameters for current setups in laboratories^12,31–33^. We observe that the only requirement for a reliable experimental observation is a reduction of initial velocity uncertainty. Importantly, we indicate that both the velocity and acceleration distributions’ maxima, stimulated by initial position uncertainty, shifts *normally* in the same direction as the velocity and acceleration mean. It is a crucial step to further accumulate such uncertainty-induced phenomena, and later use them in the aforementioned applications.

## Results

### Underdamped, overdamped and deterministic nonlinear dynamics in cubic potential

To understand the low-pressure nonlinear effects, we must distinguish them from the already measured high-pressure overdamped limit^27,30^, and aim to achieve them close to the zero-damping deterministic limit. First, we describe the properties of the stochastic motion of an underdamped Brownian particle in the unstable cubic potential. Second, we explore the high pressure limit in comparison to the over-damped approximation^27,30^, and low pressure limit with comparison to the zero-damping deterministic approximation. The dynamics of the damped Brownian particle in the cubic potential $V(x)={Kx{^3}}/3$ is described by the following Langevin equation

1 $$\begin{aligned} \ddot{x} + \gamma {\dot{x}} + \kappa x^2 = \sqrt{2\frac{k_B T\gamma }{m}}\xi (t), \qquad \langle \xi (t) \rangle =0, \langle \xi (t) \xi (t')\rangle =\delta (t-t') \end{aligned}$$

where $\kappa =K/m$ is the normalised cubic potential stiffness, $\gamma =\Gamma /m$ is the medium damping with $\Gamma$ the drag coefficient of the medium (e.g. air at low pressure), *T* is the absolute temperature, $k_B$ is the Boltzmann constant, and $F^{fluct}=\sqrt{2 k_B T\Gamma }\xi (t)$ is the broadband Markov Langevin force, uncorrelated in time with zero mean and variance given by the fluctuation-dissipation theorem $\langle F^{fluct} \rangle =0$, $\langle F^{fluct} (t) F^{fluct}(t')\rangle =\sqrt{2k_B T\gamma }\delta (t-t')$. For the short transient dynamics below the re-heating times, the uncertainty of the initial position $\sigma _{x_0}^2$ is used, instead of the thermal noise, to induce the shift with well specified ratio to the standard deviation. We use Eq. (1) for all stochastic simulations, and also to present all the figures. The details of the simulation, comprising the re-scaling of Eq. (1) to allow usage of real experimental numbers, are described in the Methods. In the high pressure limit, the instantaneous velocity and acceleration require very fast measurement to be observed in the ballistic regime^11^, therefore we use the *averaged* velocity ${\bar{v}} = \Delta x/ \Delta t$, and *averaged* acceleration ${\bar{a}}=\Delta {\bar{v}}/\Delta t$ routinely measured in high pressure experiments. The time $\Delta t$ has been adjusted to be tenfold the time-step *dt* to allow the computation of the averaged quantities. The details of the time scales, and convergence are presented in the Methods. Considering that ${\bar{v}}$ and ${\bar{a}}$ are still stochastic quantities, we further characterise their average motion by analysing their means, $\langle {\bar{v}} \rangle$, and $\langle {\bar{a}} \rangle$, and standard deviations, $\sigma _{{\bar{v}}}$, and $\sigma _{{\bar{a}}}$.

In the high pressure limit, Eq. (1) can be approximated by the over-damped equation of motion^28^,

2 $$\begin{aligned} {\dot{x}} = -\frac{K}{\Gamma } x^2 + \sqrt{2\frac{k_B T}{\Gamma }}\xi (t), \end{aligned}$$

comprising a change of mean position $\langle x(t) \rangle \approx -(K \sigma _{x_0}^2 t + 2K k_B T t^2\Gamma ^{-1})/\Gamma$, for initial Gaussian position distribution with $\langle x_0 \rangle =0$, evolving with standard deviation $\sigma _x (t) \approx \sqrt{\sigma _{x_0}^2 + 8 (K/\Gamma )^2\sigma _{x_0}^4 t^2 + 2 k_BT t/\Gamma }$, and generating a $SNR_x \approx (K^2\sigma _{x_0}^2 t^2 + K^2k_BT t^3\Gamma ^{-1})/\Gamma$^28^. The direct integration of Eqs. (1), (2) unveils short transient uncertainty-induced effects experimentally measurable, beyond linearised systems. The Gaussian statistics of the initial state, however, allows to describe the non trivial effect using only first and second Gaussian moment with different powers for different nonlinearities.

**Figure 1:**
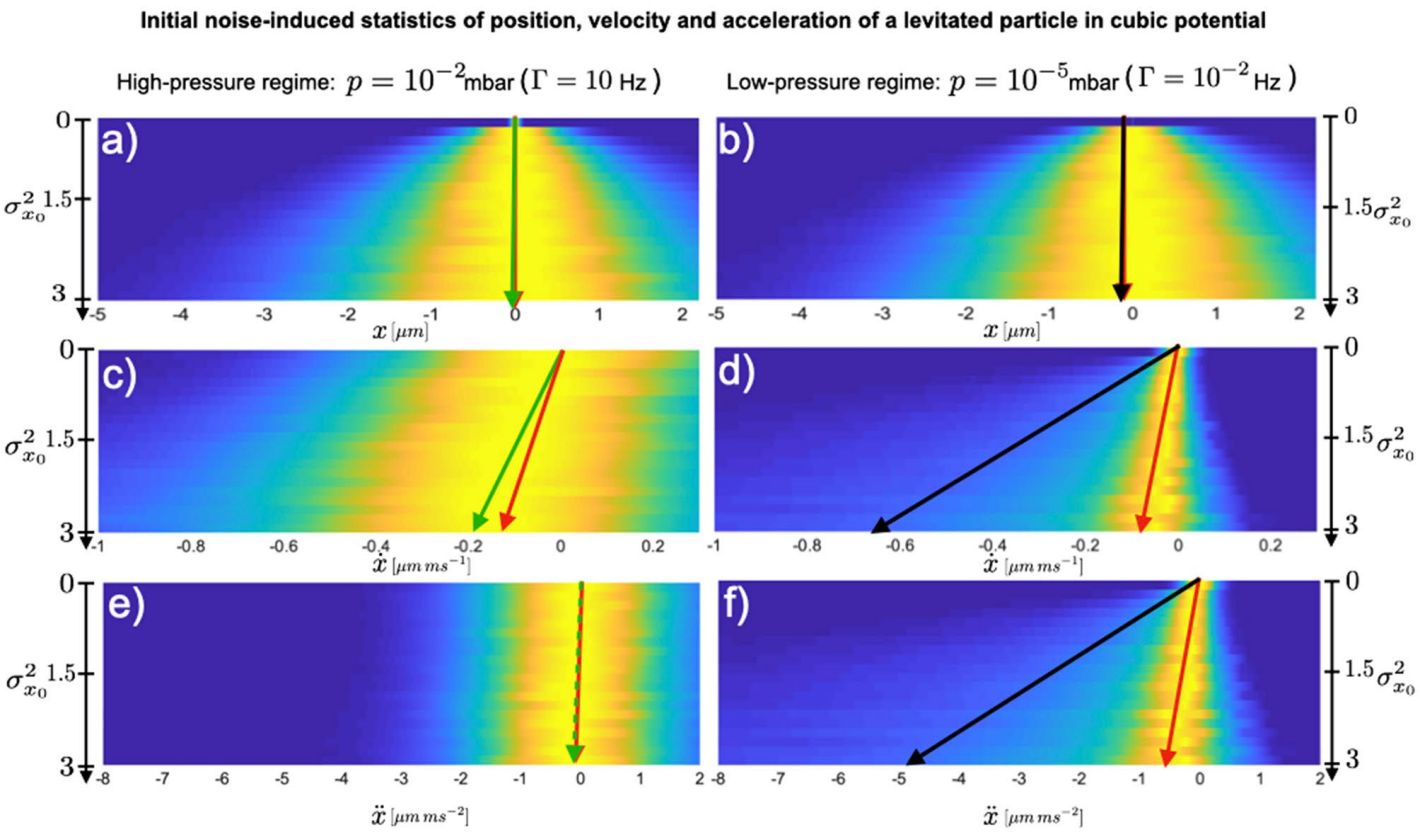
Uncertainty-induced position, velocity and acceleration statistics of a levitated particle in cubic potential, for high pressure limit, corresponding to a pressure of $p=10^{-2}$ mbar, (left column) and low pressure limit, corresponding to a pressure of $p=10^{-5}$ mbar (right column). At initial time $t=0$ ms, the Gaussian distributions of particle position has $\langle x_0 \rangle =0$, while $\sigma _{x_0}^2$ triggers the nonlinear dynamics. In all cases, the Gaussian distribution of particle instantaneous speed has $\langle {\dot{x}}_0 \rangle = 0$ and $\sigma _{{\dot{x}}_0}^2 =0$. The initial position and speed are statistically independent. In the top panel, for both high pressure limit **(a)**, and low pressure limit **(b)**, the mean of particle position $\langle x \rangle$ (green, black) does not develop at short time scales, with increasing initial position uncertainty $\sigma _{x_0}$, but its standard deviation $\sigma _x$ increases with it (green-blue halo). The maximum of position distribution $x_{max}$ (red) does not develop either with increasing initial position uncertainty. In the middle panel, for the high pressure limit **(c)**, the shift of the maximum of average velocity ${\bar{v}} = \Delta x/\Delta t$ distribution (red) increases alongside the mean of average speed $\langle {\bar{v}} \rangle$ (green). While the maximum of instantaneous velocity ${\dot{x}} =dx/dt$ distribution, in the low pressure regime **(d)** does not increase compared to its high pressure counterpart, the mean of instantaneous speed $\langle {\dot{x}} \rangle$ (black), produces a larger uncertainty-induced shift than in the high pressure regime (green). Simultaneously, the standard deviation $\sigma _{{\dot{x}}}$ (green-blue halo) and the uncertainty around the maximum $\sigma _{{\dot{x}}_{max}}$ are significantly reduced (yellow area). The bottom panel shows the evolution of particle’s acceleration statistics in both high pressure **(e)** and low pressure **(f)** limits. For the high pressure limit **(e)** neither the maximum of average acceleration ${\bar{a}} = \Delta {\dot{x}} / \Delta t$ distribution (red), nor the mean of averaged acceleration (green), $\langle {\bar{a}} \rangle$, display any uncertainty-induced shift. On the other hand, in the low pressure limit **(f)**, the statistics exhibits a substantial uncertainty-induced shift in the maximum of instantaneous acceleration $\ddot{x} = d{\dot{x}}/dt$ distribution (red) alongside the mean of instantaneous acceleration $\langle \ddot{x} \rangle$ (black). To generate all the density plots, Eq. (1) has been simulated using $\kappa =6k_BT \upmu \mathrm{m}^{-3}\, \mathrm{kg}^{-1}$,$T=300$ K, $t=0.1$ ms, $dt=2\times 10^{-5}$ ms. $N_t=10^4$ trajectories where generated with 5000 samples each. To calculate the instantaneous quantities, ${\dot{x}}=dx/dt$, $\ddot{x}=d{\dot{x}}/dt$, the time interval used is given by the time-step $dt=2\times 10^{-5}$ ms, whereas for the average quantities ${\bar{v}}=\Delta x/ \Delta t$, ${\bar{a}}=\Delta v/ \Delta t$, the time interval, multiple of the time-step, has been used $\Delta t= 10\times dt$.

At the time scale of this experiment $t=0.1$ ms, three orders of magnitude shorter than the previous overdamped experiments $t=1$s^29,30^, the mean $\langle x(t) \rangle \approx 0$ and standard deviation $\sigma _{x}(t) \approx \sigma _{x_0}$ of position do not change in time, resulting in a negligible $SNR_x$ as can be observed in Fig.1 for the high pressure regime (a). However, the average velocity $\langle {\bar{v}} \rangle$ already feels the initial uncertainty evolving linearly with the latter as shown in Fig. 1c by the green line. The statistics of the average velocity ${\bar{v}}$, for short time $\Delta t$ and Gaussian position and velocity distributions with $\langle x_0 \rangle =0$ and $\langle {\bar{v}}_0 \rangle =0$, approaches

3 $$\begin{aligned} \langle {\bar{v}} \rangle&= \left\langle \frac{\Delta x}{\Delta t} \right\rangle \approx -\frac{K}{\Gamma }\sigma _{x_0}^2, \qquad \sigma _{{\bar{v}}} \approx \sqrt{2 \left( \frac{K}{\Gamma }\right) ^2\sigma _{x_0}^4 + \frac{2k_BT}{\Gamma }}, \qquad SNR_{{\bar{v}}} = \frac{|\langle {\bar{v}} \rangle |}{\sigma _{{\bar{v}}}} \approx \frac{1}{\sqrt{2\left( 1+ k_BT \Gamma K^{-1} \sigma _{x_0}^{-2}\right) }}. \end{aligned}$$

**Figure 2:**
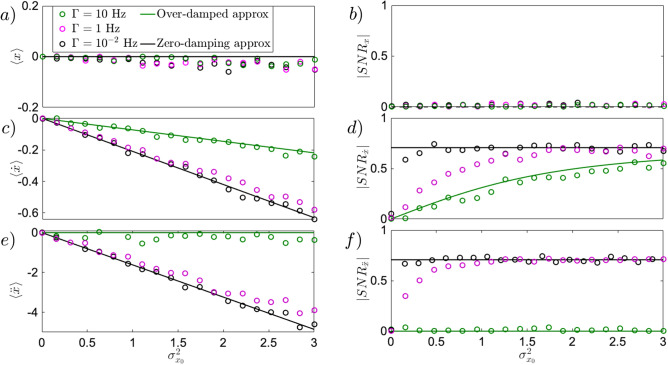
Uncertainty-induced effect for initially steady particle in position *x* **(a,b)**, instantaneous velocity ${\dot{x}}$ **(c,d)**, and acceleration $\ddot{x}$ **(e,f)**, driven by variance $\sigma _{x_0}^2$ of initial position. All simulations (dots) have been performed based on Eq. (1). In the top panel, for a short time scale up to $t=0.1$ ms, the position statistics is trivial for all damping values, as the mean $\langle x \rangle$ **(a)** does not develop with increasing initial uncertainty of position. Similarly, the $SNR_x$ **(b)**, is dominated by the noise spread and therefore vanishes. In the middle panel, **(c)**, the evolution of the mean of instantaneous velocity is displayed for different values of damping. At low damping, $\Gamma =10^{-2}$ Hz (black dots), the deterministic limit (black line), as derived in Eq. (6), can be reached. On the other hand, for higher damping, namely $\Gamma =10$ Hz (green dots), the over-damped limit (green line), described by the mean of average velocity $\langle {\bar{v}} \rangle \approx - (K/\Gamma )\sigma _{x_0}^2$ is approached. The middle case of $\Gamma =1$ Hz (purple dots), shows the sensitivity of mean of instantaneous velocity, $\langle {\dot{x}} \rangle$, to the damping coefficient $\gamma$. Simultaneously, in **(d)** the evolution of the $SNR_{{\dot{x}}}$ depicts the role of the environmental temperature *T* for different pressures, showing a regime dominated by the latter (purple dots) denoted by a linear increase of the $SNR_{{\dot{x}}}$ as discussed in Eq. (9). In the bottom panel, **(e)**, the mean of instantaneous acceleration is depicted for different pressures. While for small damping $\Gamma =10^{-2}$ Hz (black dots), the shift of mean instantaneous acceleration, $\langle \ddot{x} \rangle$, is visible and close to the deterministic limit (black line), given in Eq. (5). It vanishes for larger damping $\Gamma =10$ Hz (green dots), where no instantaneous acceleration exists, **(f)** depicts, by contrast, the role of the environmental temperature *T* recognisable in the $SNR_{\ddot{x}}$ by the linear dependency in $\sigma _{x_0}^2$ of initial position, to be less present even at damping $\Gamma =1$ Hz, in comparison to the case for velocity, as shown in Eq. (10). Eq. (1) has been simulated using $\kappa =6k_BT \mu m^{-3}Kg^{-1}$,$T=300$ K (ambient temperature), $\langle x_0 \rangle =0$, $\langle {\dot{x}}_0 \rangle =0$, $\sigma _{{\dot{x}}_0}=0$, $t=0.1$ ms, $dt=2\times 10^{-5}$ ms. $10^4$ trajectories where generated with 5000 samples each. To compute the average quantities, ${\bar{v}}$, ${\bar{a}}$, the multiple time-step has been used, $\delta t = 10 \times dt$.

For small environmental temperature, $k_B T \ll K^2/\Gamma$, Eq. (3) generates a constant $SNR_{{\bar{v}}} \approx 1/\sqrt{2}$. On the other hand, in the experiments at room temperature and $\Gamma =10$ Hz^29,30^ the second term in the standard deviation of average velocity $\sigma _{{\bar{v}}}$ reduces the $SNR_{{\bar{v}}}$ to that of Eq. (3). The latter can be qualitatively observed in Fig.1c by the non constant azure halo as a function of initial position uncertainty. However, velocity uncertainty does not increase as quickly as in particle position, for the same short time scale. The statistics of the average acceleration ${\bar{a}}$, as shown in Fig.1e, do not change neither for the mean $\langle {\bar{a}} \rangle$ (green), nor for the standard deviation $\sigma _{{\bar{a}}}$ (blue–green halo) for the short time dynamics and range of parameters we are interested in, and their approximate characterisation is therefore omitted. By numerically simulating Eq. (1) for values of $\Gamma \ge 10$ Hz, the position and average velocity statistics, obtained from Eq. (2) for the over-damped approximation, can be retrieved fully. A quantitative comparison is shown in Fig. 2(a,c, green dots).

The aim of the low-pressure regime is to (i) reach the statistics of *instantaneous* speed, as compared to average speed in Eq. (3), and (ii) obtain *instantaneous* acceleration statistics, both not achievable in the high pressure regime without very fast measurements. We consider such a short-time regime characterised by no change in initial particle position, as depicted in Fig. 1. For better and clear visualisation, the scale bar for increasing initial uncertainty $\sigma _{x_0}^2$ changes within, while conserving the information about the profile of the probability density, up to the normalisation factor.

Such short transient regime when the particle does not move defines the time scale of such effect.

The goal in this regime is to reach the ideal classical limit of the zero-pressure regime, approaching deterministic dynamics for $\Gamma =0$, where Eq. (1) becomes

4 $$\begin{aligned} \ddot{x} + \kappa x^2=0, \end{aligned}$$

yielding deterministic trajectories from initial position and velocity statistics. In this nonlinear ballistic regime, the Gaussian initial distribution of particle speed has $\langle {\dot{x}}_0 \rangle = 0$ and $\sigma _{{\dot{x}}_0}^2 =0$. The initial position and speed are statistically independent. In such regime, the initial position statistics is the only thermal energy resource that can be used in the nonlinear ballistics. For short time dynamics, from $\langle x_0 \rangle =0$, the quantitative description of the zero-damping approximation for instantaneous acceleration gives

5 $$\begin{aligned} \langle \ddot{x} \rangle \approx -\kappa \sigma ^2_{x_0}, \qquad \sigma _{\ddot{x}} \approx \sqrt{2}\kappa \sigma ^2_{x_0}, \qquad SNR_{\ddot{x}} = \frac{|\langle \ddot{x} \rangle |}{\sigma _{\ddot{x}}} \approx \frac{1}{\sqrt{2}}. \end{aligned}$$

Notice that both mean and standard deviation are advantageously independent of initial speed statistics. This helps to distinguish the nonlinear ballistics in the experiment. The evolution of mean instantaneous acceleration $\langle \ddot{x} \rangle$, and its standard deviation $\sigma _{\ddot{x}}$, grow comparably with $\sigma _{x_0}$ keeping a constant $SNR_{\ddot{x}}\approx 1/\sqrt{2}$. In Fig.2e,f, Eq. (1) has been simulated (black dots), for damping $\Gamma =10^{-2}$ Hz, to generate $\langle \ddot{x} \rangle$, and $SNR_{\ddot{x}}$. At such small pressure, the dominant dynamics observed can be already described by the zero-damping approximation introduced in Eq. (4), resulting in a linear evolution of $\langle \ddot{x} \rangle$ with $\sigma _{x_0}^2$ as showed in Eq. (5) (black line). Simultaneously, the $SNR_{\ddot{x}}$ (black dots) generated with Eq. (1), converges quickly to the zero-damping approximation depicted in Eq. (4), resulting in a constant $SNR_{\ddot{x}}$ as obtained in Eq. (5). By formally integrating Eq. (4) ${\dot{x}}(t) \approx {\dot{x}}_0 - \kappa \int _0^t x_0^2(t') dt'$ with $\langle x_0 \rangle =0$, the short-time evolution of instantaneous velocity unfolds as

6 $$\begin{aligned} \langle {\dot{x}}(t) \rangle \approx - \kappa \sigma ^2_{x_0} t, \qquad \sigma _{{\dot{x}}}(t) \approx \sqrt{2} \kappa \sigma ^2_{x_0} t, \qquad SNR_{{\dot{x}}}(t) = \frac{|\langle {\dot{x}}(t) \rangle |}{\sigma _{{\dot{x}}}(t)} \approx \frac{1}{\sqrt{2}}. \end{aligned}$$

considering the initial conditions $\langle {\dot{x}}_0 \rangle =\sigma _{{\dot{x}}_0}^2=0$. The mean $\langle {\dot{x}}(t)\rangle$ of instantaneous velocity evolves linearly with initial position variance $\sigma _{x_0}^2$, growing comparably with its standard deviation $\sigma _{{\dot{x}}}(t)$, ultimately leading to the constant $SNR_{{\dot{x}}}\approx 1/\sqrt{2}$ shown in Eq. (6). Simultaneously, by second time formally integrating Eq. (4) with $\langle x_0 \rangle =0$, the position approaches $x(t) \approx x_0 + \int _0^{t}{\dot{x}}_0(t') dt' -\int _0^{t}\int _0^{t'}\kappa x_0^2(t'') dt''dt'$, while its statistics for short time dynamics evolves as

7 $$\begin{aligned} \langle x(t) \rangle \approx - \frac{1}{2}\kappa \sigma _{x_0}^2t^2, \qquad \sigma _{x}(t) \approx \sqrt{\sigma _{x_0}^2 + \frac{1}{2}\kappa ^2t^4\sigma _{x_0}^4}, \qquad SNR_x(t) = \frac{|\langle x \rangle |}{\sigma _{x}} \approx \frac{1}{\sqrt{2\left( 1+ 2 k^{-2} \sigma _{x_0}^{-2} t^{-4}\right) }}. \end{aligned}$$

For the large time scale, starting at $t=0.9$ ms, the mean position evolves as in Eq. (7), while the standard deviation is dominated by the second term, ultimately leading to a constant $SNR_x \approx 1/\sqrt{2}$. At this scale, the dynamics already makes the particle moving farther by initial position uncertainty. At the short time scale, $t=0.1$ ms however, the position at average does not move, while its standard deviation remains equal to $\sigma _{x_0}$. The resulting uncertainty-induced dynamics produces a $SNR_x \approx 0$. At such a short time scale, the result is similar to the over-damped regime described by  (2). This behavior can be observed in Fig.1b by the black arrow for the mean position, and with the azure halo for the standard deviation. The behavior of the $SNR_x$ can be observed in Fig.2(b, black line), holding true for small pressure (black dots), as well as for high pressure (green dots).

### Robustness of the uncertainty-induced instantaneous speed and acceleration

#### Role of environmental temperature *T*

The instantaneous speed and acceleration stimulated by the initial position uncertainty, can be affected by room temperature of external environment. Generalising the above results for non-zero damping $\gamma$, and environmental temperature *T*, assuming initial Gaussian distribution both in *x* and ${\dot{x}}$ with $\langle x_0 \rangle =\langle {\dot{x}}_0 \rangle =0$, and vanishing variance $\sigma _{{\dot{x}}_0}^2=0$, the role of environmental noise can be discussed using the approximate formulae obtained from Eq. (1)

8 $$\begin{aligned} \langle x(t) \rangle&\approx - \frac{1}{2}\kappa \sigma _{x_0}^2t^2, \quad \sigma _{x}(t) \approx \sqrt{\sigma _{x_0}^2 + \frac{2 k_BT\gamma }{3m}t^3 + \frac{1}{2} \kappa ^2 \sigma _{x_0}^4 t^4}, \quad SNR_x(t) = \frac{|\langle x \rangle |}{\sigma _{x}} \approx \frac{1}{\sqrt{2 \left( 1 + \frac{2 k_B T \gamma }{3 m \kappa ^2 \sigma _{x_0}^4 t} + \frac{2}{\kappa ^2 \sigma _{x_0}^2 t^4} \right) }} \end{aligned}$$

9 $$\begin{aligned} \langle {\dot{x}}(t) \rangle&\approx - \kappa \sigma ^2_{x_0} t, \qquad \sigma _{{\dot{x}}}(t) \approx \sqrt{2 \kappa ^2 \sigma ^4_{x_0} t^2 + 2\frac{k_BT \gamma }{m}t}, \qquad \qquad SNR_{{\dot{x}}}(t) =\frac{|\langle {\dot{x}} \rangle |}{\sigma _{{\dot{x}}}} \approx \frac{1}{\sqrt{2 \left( 1+ \frac{k_B T\gamma }{m \kappa ^2 \sigma _{x_0}^4 t} \right) }}, \end{aligned}$$

10 $$\begin{aligned} \langle \ddot{x} (t) \rangle&\approx -\kappa \sigma ^2_{x_0}, \qquad \sigma _{\ddot{x}}(t) \approx \sqrt{2\kappa ^2\sigma ^4_{x_0} + 2\frac{k_BT \gamma }{m}}, \qquad \quad \qquad \, \, SNR_{\ddot{x}}(t) = \frac{|\langle \ddot{x} \rangle |}{\sigma _{\ddot{x}}} \approx \frac{1}{\sqrt{2 \left( 1+ \frac{k_B T \gamma }{m\kappa ^2 \sigma _{x_0}^4}\right) }} . \end{aligned}$$

The main significant difference, noticeable from Eqs. (8) to  (10), is the time scale at which the environmental temperature *T* becomes relevant. Notice how advantageously the $SNR_{\ddot{x}}$ of Eq. (10) is time independent, compared to Eqs. (8), and  (9). In Eq. (8) for position statistics, *T* plays a negligible role for short time scales (up to $t=0.1$ ms), as the $SNR_x \approx 0$ is independent of the chosen *T*, as visible from Fig.2b for the range of $\Gamma$ from overdamped to zero-damping limit. For longer time scales (from $t=0.3$ ms) the environmental temperature competes against the uncertainty-induced effect when $T \ge 3 m \kappa ^2 t \sigma _{x_0}^4/(4 k_B \gamma )$ ($T \approx 800\sigma _{x_0}^4$ K, for $\Gamma =10^{-2}$ Hz), realising a growing $SNR_x$ slowly converging to $SNR_x=1/\sqrt{2}$ for the higher environmental temperature *T*. Given the high temperature requirement, such regime is not visible for the parameters of the experimental set-up we used^12^. In Eq. (9), the standard deviation of instantaneous velocity $\sigma _{{\dot{x}}}(t)$ is not affected by ambient temperature $T=300$ K at low pressure, as the required condition to reduce the $SNR_{{\dot{x}}}(t)$ is $T \ge m \kappa ^2 \sigma _{x_0}^4 t/k_B \gamma$ ($T \approx 125\times 10^2 \sigma _{x_0}^4$ for $\Gamma =10^{-2}$ Hz (black dots). Differently is the case at higher pressures, $\Gamma =1$ Hz (Fig.2d, purple dots), where the condition softens to $T \approx 140 \sigma _{x_0}^4$, reducing the $SNR_{{\dot{x}}}(t)$ at small initial uncertainty $\sigma _{x_0}^2$. The condition, for environmental temperature *T*, in the standard deviation of instantaneous acceleration in Eq. (10) $\sigma _{\ddot{x}}$, is much harder to fulfill. As can be glimpsed from Fig.2f, for the low pressure limit (black dots) the environmental noise is negligible even at ambient temperature $T=300$ K, leaving the $SNR_{\ddot{x}}(t) \approx 1/\sqrt{2}$ unmodified. To be able to witness the effect of the environmental noise at such low pressures $\Gamma =10^{-2}$ Hz, Eq. (10) provides a condition $T \ge m \kappa ^2 \sigma _{x_0}^4/k_B \gamma$ ($T \approx 150\times 10^2 \sigma _{x_0}^4$K, for $\Gamma =10^{-2}$ Hz) showing the high temperature requirement, index of high robustness against environmental noise. For higher pressures, $\Gamma =1$ Hz (purple dots), the condition softens to $T \approx 144\times 10^2 \sigma _{x_0}^4$ K, allowing, for small $\sigma _{x_0}^2$ to witness a reduction of the $SNR_{\ddot{x}}(t)$ (purple dots).

#### Role of initial particle velocity

Thus far we have considered the case of an initially steady particle with $\langle x_0 \rangle =0$. Assuming we have infinitely precise control over the choice of initial speed, $\sigma _{{\dot{x}}_0}^2=0$, one can observe the uncertainty-induced effect for moving particles, rendering the experimental test broad. Generalising Eq. (7) for non-zero $\langle {\dot{x}}_0 \rangle$, and introducing $\Delta x = x(t) - {\dot{x}}_0 t$, to exclude deterministic position change, one obtains

11 $$\begin{aligned} \langle \Delta x \rangle \approx - \frac{\kappa \sigma _{x_0}^2 t^2}{2}, \qquad \sigma _{\Delta x} \approx \sqrt{\sigma _{x_0}^2 + \frac{k^2 t^4 \sigma _{x_0}^4}{2}}, \qquad SNR_{\Delta x} = \frac{|\Delta x|}{\sigma _{\Delta x}} \approx \frac{1}{\sqrt{2\left( 1+ 2 k^{-2} \sigma _{x_0}^{-2} t^{-4}\right) }}. \end{aligned}$$

**Figure 3:**
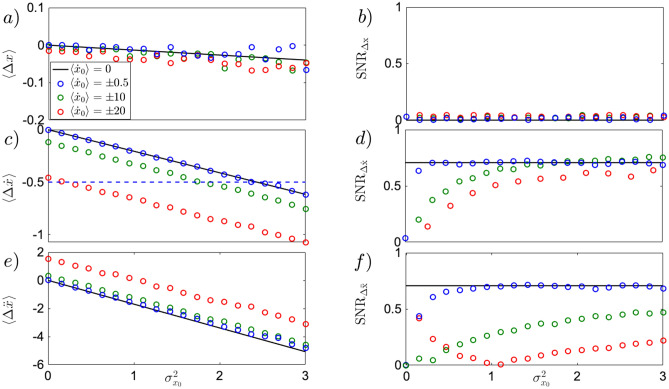
Uncertainty-induced effects for initially moving particle in position statistics **(a,b)**, instantaneous velocity statistics **(c,d)**, and instantaneous acceleration statistics **(e,f)**. All simulations (dots) have been performed based on Eq. (1) using $\sigma _{{\dot{x}}_0}^2=0$. For mean particle position **(a)** the dynamics remains unchanged from that of Eq. (11) (black line) independent of the chosen initial velocity $\langle {\dot{x}}_0 \rangle$, while the $SNR_{\Delta x}$ (**b**, dots) remains small (close to zero), as the standard deviation increases faster than the mean. For mean instantaneous velocity (**c,d**), the approximation introduced in Eq. (12) (black line) holds true for values of initial velocity up to $\langle {\dot{x}}_0 \rangle = \pm 5$. Within this frame, stochastic speeding (for $\langle {\dot{x}}_0 \rangle > 0$) and stochastic breaking (for $\langle {\dot{x}}_0 \rangle < 0$) are witnessed when $\langle \Delta {\dot{x}} \rangle = - |\langle {\dot{x}}_0 \rangle |$. Specifically, for $\langle {\dot{x}}_0 \rangle = \pm 0.5$ (blue dots), the initial position uncertainty induced stochastic speeding/breaking is met at values of $\langle \Delta {\dot{x}} \rangle = -0.5$ (blue dashed line). For values $| \langle {\dot{x}}_0 \rangle | > 5$ (green, and red dots) the dynamics can no longer be described by Eq. (12), as higher order nonlinear terms become relevant. As a result, the slope remains similar to that of the black line, but the curve is shifted to a non-zero value at $\sigma _{x_0}^2 =0$. The resulting $SNR_{\Delta {\dot{x}}}$ display a slower convergence to the black line as the initial velocity becomes larger in magnitude. Particle acceleration (**e,f**) displays a solid robustness against the initial velocity $\langle {\dot{x}}_0 \rangle < 20$ in both mean (**e**), and $SNR_{\Delta \ddot{x}}$ (**f**). However, for values of $\langle {\dot{x}}_0 \rangle \ge 20$ (red dots), the evolution of mean instantaneous acceleration $\langle \Delta \ddot{x} \rangle$ deviates from the approximation of Eq. (13) (black line). In this regime, the $SNR_{\Delta \ddot{x}}$ presents an initial decrease, index of highly unstable dynamics, followed by a slow convergence towards the black line. Eq. (1) has been simulated using $\kappa =6 k_BT\upmu m^{-3}Kg^{-1}$, $T=300$ K, $\Gamma =10^{-2}$ Hz, $t=0.1$ ms, $dt=2\times 10^{-5}$ ms. $10^4$ trajectories where generated with 5000 samples each.

As visible from Fig.3a,b, Eq. (11) holds true for particles moving at different initial speed (blue, green and red dots), showing that even quickly moving particles at the considered short time scale $t=0.1$ ms do not move on average.

For moving particles, the equation for instantaneous velocity reads ${\dot{x}}(t) = {\dot{x}}_0 - k x_0^2t$. From the latter, the velocity difference $\Delta {\dot{x}} = {\dot{x}}(t) - {\dot{x}}_0$ is introduced, and its statistical evaluation comes as follows

12 $$\begin{aligned} \langle \Delta {\dot{x}} \rangle \approx - \kappa \sigma _{x_0}^2 t, \qquad \sigma _{\Delta {\dot{x}}} \approx \sqrt{2} \kappa \sigma _{x_0}^2 t, \qquad SNR_{\Delta {\dot{x}}} = \frac{|\langle \Delta {\dot{x}} \rangle |}{\sigma _{\Delta {\dot{x}}}} \approx \frac{1}{\sqrt{2}}. \end{aligned}$$

As noticeable from Fig.3c,d, the mean instantaneous velocity of Eq. (12) holds true only for values of initial velocities $\langle {\dot{x}}_0 \rangle = \pm 0.5$ (blue dots). However, for particles initially moving at higher speed (green and red dots), the approximation in Eq. (12) fails to describe the motion now modified by higher order nonlinear terms, as the particle obtains more negative mean instantaneous velocity for smaller $\sigma _{x_0}^2$. This high order nonlinear effects can only be investigated by numerical stochastic simulations. The $SNR_{{\dot{x}}}$ (middle panel, right column), although affected by the change in initial velocity (green and red dots), for larger $\sigma _{x_0}^2$ converges to its constant value $SNR_{{\dot{x}}}=1/\sqrt{2}$ of Eq. (12).

To visualise the effect of initial velocity ${\dot{x}}_0$ on instantaneous acceleration $\ddot{x}$, one needs to explore the second order approximation to Eq. (4). We rewrite Eq. (4) to a set of dynamical equations for the position *x* and speed ${\dot{x}}$. Substituting a short-time solution $x \approx x_0 +{\dot{x}}_0 t$ of the equation for the position *x*, to the right side of the equation for the speed ${\dot{x}}$ , we obtain a solution for the acceleration $\ddot{x}(t) \approx -\kappa (x_0^2 +2 x_0 {\dot{x}}_0 t +{\dot{x}}_0^2 t^2)$. Introducing the quantity $\Delta \ddot{x} = \ddot{x}(t) + \kappa {\dot{x}}_0^2t^2$, a pure effect of $\sigma _{x_0}^2$ on acceleration can be described, for $\langle x_0 \rangle =0$, non vanishing $\langle {\dot{x}}_0 \rangle$, and $\sigma _{{\dot{x}}_0}^2=0$ as follows

13 $$\begin{aligned} \langle \Delta \ddot{x} \rangle \approx - \kappa \sigma _{x_0}^2, \qquad \sigma _{\Delta \ddot{x}} \approx \sqrt{2} \kappa \sigma _{x_0}^2, \qquad SNR_{\Delta \ddot{x}} = \frac{|\langle \Delta \ddot{x} \rangle |}{\sigma _{\Delta \ddot{x}}} \approx \frac{1}{\sqrt{2}}. \end{aligned}$$

Evidently, in Fig.3e,f, for a particle with small non-zero initial speed ${\dot{x}}_0$, the acceleration does not deviate from the approximation introduced in Eq. (13), but when ${\dot{x}}_0$ increases, the mean acceleration gets closer to zero and $SNR_{\Delta \ddot{x}}$ approaches $1/\sqrt{2}$ much slower for larger $\sigma _{x_0}^2$, as visible in Fig.3(e,f, green). For even larger initial speed (red dots), the particle obtains less negative average acceleration for smaller $\sigma _{x_0}^2$, producing an initially decreasing $SNR_{\Delta \ddot{x}}$. Decreasing the time scale tenfold $t=0.01$ ms brings the red dots closer to the zero-damping approximation (black line), indicating that the shift is generated by higher order nonlinear terms beyond the approximate result introduced in Eq. (13). The larger $\sigma _{x_0}^2$, the more the particle accelerates, resulting in an increase of the $SNR_{\Delta \ddot{x}}$, slowly approaching $1/\sqrt{2}$.

Fig.3 demonstrates that the uncertainty-induced shift of particle position (a,b), instantaneous velocity (c,d) and instantaneous acceleration (e,f) can be observed also for slowly and deterministically moving particles (black, and blue, and green dots). Moreover, for $\langle {\dot{x}}_0 \rangle >0$ stochastic breaking induced by initial uncertainty can be witnessed where the particle breaks at average $\langle {\dot{x}} \rangle =0$ for sufficiently large initial position uncertainty $\sigma _{x_0}^2$. Similarly for $\langle {\dot{x}}_0 \rangle <0$ stochastic speeding is observed, where the particle gains velocity for sufficiently large initial position uncertainty $\sigma _{x_0}^2$. The latter can be observed even during the short time period, and it is noticeable by the sign flip of mean instantaneous velocity $\langle \Delta {\dot{x}}_0 \rangle = - |{\dot{x}}_0|$ (Fig.3c,d, dashed blue line).

#### Sensitivity to velocity uncertainty

Complementary to the previous section, here we investigate how sensitive the uncertainty induced effect is to an increase of initial velocity uncertainty $\sigma _{{\dot{x}}_0}^2$, for strictly zero initial velocity $\langle {\dot{x}}_0 \rangle =0$, and $\langle x_0 \rangle =0$. Assuming negligible $\gamma$, as witnessed already in Fig.2 for the short time dynamics, Eq. (1) can be studied to describe the effect of initial statistics of velocity. Due to non-zero initial velocity uncertainty, Eqs. (5)–(7) modify to

14 $$\begin{aligned} \langle x(t) \rangle&\approx -\frac{\kappa \sigma _{x_0}^2 t^2}{2}, \qquad \quad \qquad \sigma _{x} \approx \sqrt{\sigma _{x_0}^2 + \sigma _{{\dot{x}}_0}^2 + \frac{1}{2}\kappa ^2 \sigma _{x_0}^4 t^4}, \qquad \quad \! \! \! \! SNR_x \approx \frac{1}{\sqrt{2}\sqrt{\frac{2\sigma _{x_0}^2 + 2\sigma _{{\dot{x}}_0}^2 t^2}{\kappa ^2 \sigma _{x_0}^4 t^4}+1}} \end{aligned}$$

15 $$\begin{aligned} \langle {\dot{x}}(t) \rangle&\approx - \kappa \sigma ^2_{x_0}t, \qquad \qquad \quad \quad \! \! \! \sigma _{{\dot{x}}} \approx \sqrt{\sigma _{{\dot{x}}_0}^2+ 2\kappa ^2t^2\sigma _{x_0}^4}, \qquad \qquad \quad \, \, SNR_{{\dot{x}}} \approx \frac{1}{\sqrt{2}\sqrt{\frac{\sigma _{{\dot{x}}_0}^2}{2\kappa ^2 \sigma _{x_0}^4 t^2}+1}} \end{aligned}$$

16 $$\begin{aligned} \langle \ddot{x}(t) \rangle&\approx -\kappa (\sigma ^2_{x_0} + \sigma _{{\dot{x}}_0}^2 t^2), \qquad \sigma _{\ddot{x}} \approx \sqrt{2 \kappa ^2(\sigma _{x_0}^4 + \sigma _{{\dot{x}}_0}^4 t^4)}, \qquad \qquad \quad \! \! SNR_{\ddot{x}} \approx \frac{\left( \frac{\sigma _{x_0}^2}{\sigma _{{\dot{x}}_0}^2} + t^2 \right) }{\sqrt{2} \sqrt{\frac{\sigma _{x_0}^4}{\sigma _{{\dot{x}}_0}^4}+t^4 }}. \end{aligned}$$

**Figure 4:**
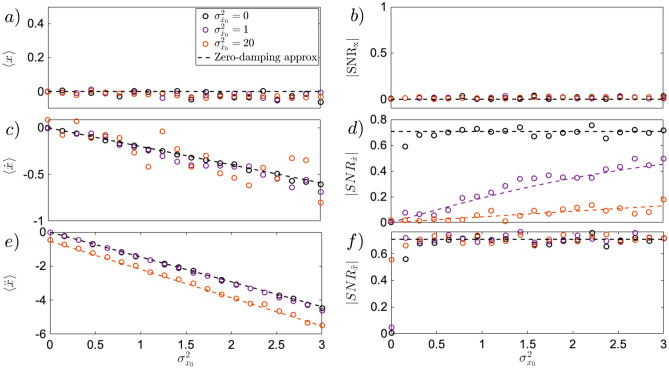
Robustness of initial uncertainty-induced effect to initial velocity uncertainty $\sigma _{{\dot{x}}_0}^2$ for position statistics **(a,b)**, instantaneous velocity statistics **(c,d)**, and instantaneous acceleration **(e,f)**. All simulations (dots) have been performed based on Eq. (1) using $\langle x_0 \rangle =\langle {\dot{x}}_0 \rangle =0$. In the top panel, for particle position, the increasing initial velocity uncertainty $\sigma _{{\dot{x}}_0}^2$ does not modify the statistics of both mean position **(a)**, and $SNR_x$ **(b)**. In the middle panel, for instantaneous velocity, the uncertainty-induced feature is still observable for increasing initial velocity uncertainty $\sigma _{{\dot{x}}_0}^2$, at the cost of high ensemble size requirements for mean instantaneous velocity computation **(c)**. The $SNR_{{\dot{x}}}$ **(d)** still shows the uncertainty induced effect, increasing with initial position uncertainty, but its statistics gets modified, according to Eq. (15) (purple and orange dots), to a linearly increased $SNR_{{\dot{x}}}$ The bottom panel, **(e,f)** for instantaneous acceleration, displays a small sensitivity to initial velocity uncertainty $\sigma _{{\dot{x}}_0}^2$. As obtained from Eq. (16), the condition the initial position uncertainty must fulfill to overthrow the initial velocity uncertainty, $\sigma _{x_0}^2 \gg \gamma \sigma _{{\dot{x}}_0}/\sqrt{2} \kappa \approx 5\times 10^{-3}\sigma _{{\dot{x}}_0}$, is quite trivial, rendering the impact of $\sigma _{{\dot{x}}_0}^2$ negligible upon the statistics of instantaneous acceleration, never reaching, for the parameters of the experiment used, the regime introduced in Eq. (16) for $SNR_{\ddot{x}}$. The case of large initial velocity uncertainty $\sigma _{{\dot{x}}_0}^2=20$ (orange dots) introduces enhancement of mean instantaneous acceleration **(e)**, that can be described by $\langle \ddot{x} \rangle \approx - \kappa (\sigma _{x_0}^2 + \sigma _{{\dot{x}}_0}^2t^2)$ as introduced in Eq. (16). Eq. (1) has been simulated using $\kappa =6k_BT\upmu \mathrm{m}^{-3}\,\mathrm{kg}^{-1}$, $T=300$ K, $\Gamma =10^{-2}$ Hz, $t=0.1$ ms, $dt=2\times 10^{-5}$ ms. $N_t=10^4$ trajectories where generated with $N=5000$ samples each.

At short time scale, the mean position $\langle x(t) \rangle$ does not move, resulting in a vanishing $SNR_x$ independent of the value of the initial velocity uncertainty, as visible in Fig.4a,b. For time larger than $t=0.3$ ms, when the nonlinear terms in Eq. (14) are prominent and $SNR_x$ approaches $1/\sqrt{2}$, the initial velocity uncertainty can modify the statistics of position if $\sigma _{{\dot{x}}_0}^2 \gg \kappa \sigma _{x_0}^2t^2/\sqrt{2}$ ($\sigma _{{\dot{x}}}^2 \approx 10^{-2} \sigma _{x_0}^2$, at $t=0.3$ ms, $\Gamma =10^{-2}$ Hz). The statistics of instantaneous velocity, as described in Eq. (15) are more affected by increasing initial velocity uncertainty at short time scales. For values of $\sigma _{{\dot{x}}_0}^2 \gg 2\kappa ^2 t^2\sigma _{x_0}^2 \approx 4\times 10^{-2}\sigma _{x_0}^2$ (at $t=0.1$ ms, $\Gamma =10^{-2}$ Hz), the statistics of $SNR_{{\dot{x}}}$ slowly approaches the constant $1/\sqrt{2}$ value, and rather approaches that of Eq. (15) as shown in Fig.4(d, purple dashed line). As can be seen directly from Eq. (16), instantaneous acceleration bears the ability to be driven both by initial, velocity uncertainty $\sigma _{{\dot{x}}_0^2}$, dominating in the long time scale, and initial position uncertainty $\sigma _{x_0^2}$, dominating in the short time scale. As can be seen in Fig.4e at timescales of $t=0.1$ ms the term $\sigma _{x_0^2}$ dominates over the dynamics (black and purple dots), retrieving the zero-damping approximation results (black dashed line). At larger values of $\sigma _{{\dot{x}}_0^2}$ (orange dots) the enhancing effects of initial velocity uncertainty becomes visible as the mean instantaneous acceleration becomes more negative than the zero-damping approximation (black dashed line). Simultaneously the $SNR_{\ddot{x}}$ (e) quickly converges to $1/\sqrt{2}$ independent of the choice of initial velocity uncertainty.

### Coherent motion of maximum of distribution

**Figure 5:**
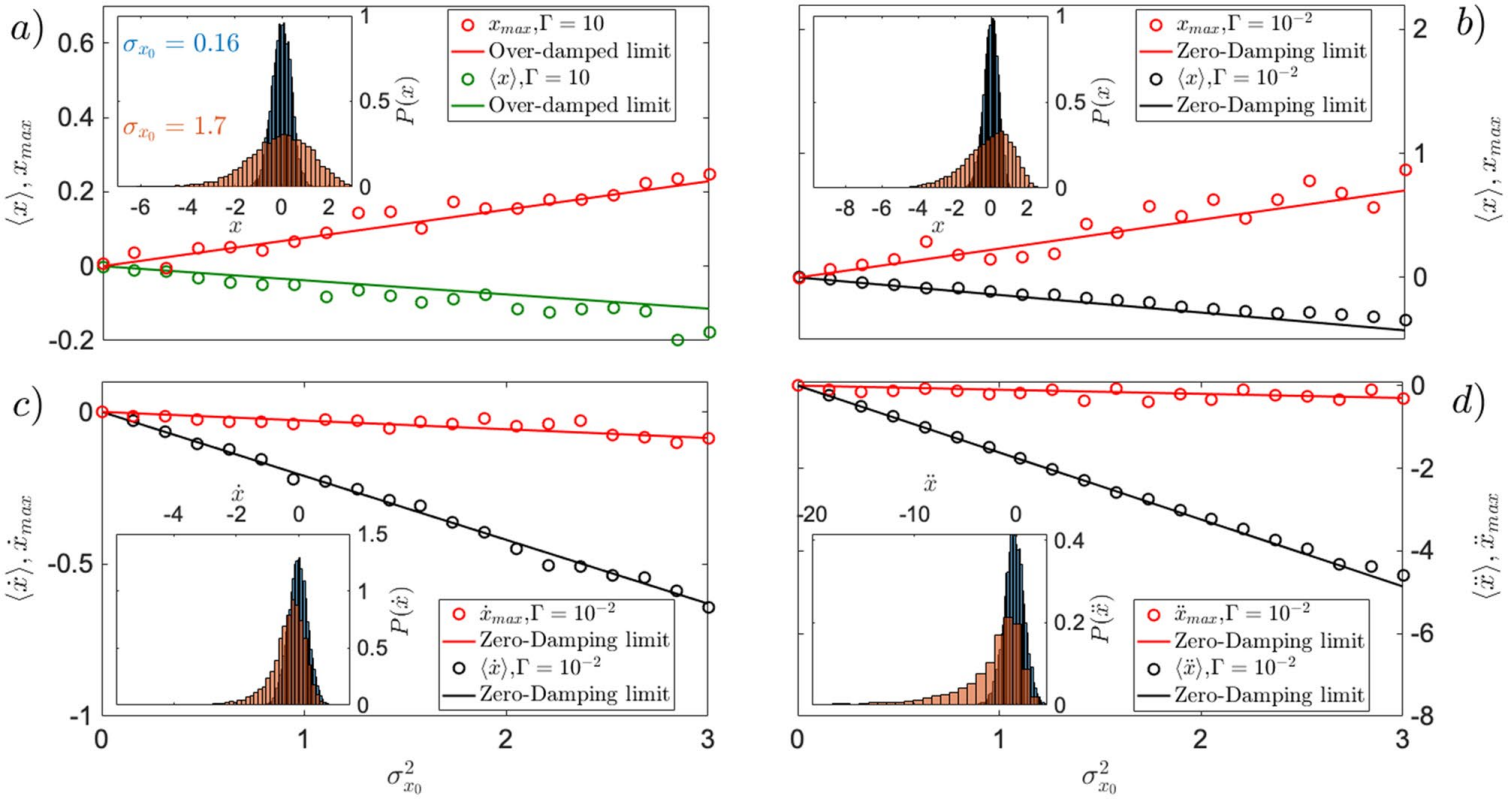
Initial uncertainty-induced shift of maximum of position $x_{max}$, for high **(a)** and low **(b)** pressure limit, maximum of instantaneous velocity ${\dot{x}}_{max}$ **(c)**, and acceleration $\ddot{x}_{max}$ **(d)**. All simulations (dots) have been performed based on Eq. (1) using $\langle x_0 \rangle =\langle {\dot{x}}_0 \rangle =0$, and $\sigma _{{\dot{x}}_0}^2=1$. The top panel highlights the atypical evolution of maximum of particle position $x_{max}$ (**a**, red dots) retrieved in^27,30^ for over-damped dynamics (**a**, red line), and witnessed again in the low pressure limit (**b**, red). To make the shift in maximum more visible, Eq. (1) has been simulated with $t=0.3$ ms. The maximum of instantaneous velocity ${\dot{x}}_{max}$, in the low pressure limit **(c)**, introduces a new effect comprising of a coherent shift of ${\dot{x}}_{max}$ (red) alongside the potential force (black) The inset, showing snapshots of the $P({\dot{x}}$ at different $\sigma _{x_0}^2$, highlights the instability (heavy tails on the left), with a clear shift of the maximum alongside. Similarly, the maximum of instantaneous acceleration $\ddot{x}_{max}$ (**d**, red), shifts coherently with its mean $\langle \ddot{x} \rangle$ (black), corroborated by the inset picture, showing the left shift of $\ddot{x}_{max}$ for different values of $\sigma _{x_0}^2$. By comparing the shift of ${\dot{x}}_{max}$, and $\ddot{x}_{max}$, we notice that the latter is comprised of a larger shift induced by initial position uncertainty $\sigma _{x_0}^2$. Moreover, comparing the $P({\dot{x}})$, and $P(\ddot{x})$ (inset **a,b**), with the *P*(*x*) (inset **c,d**), we notice the absence of light tail in the former, that is visible in position^27^. To produce the figure on the bottom panel, Eq. (1) has been simulated with $t=0.1$ ms. The other parameters used to produce this graph from Eq. (1) are $\kappa =6k_BT \upmu \mathrm{m}^{-3} kg^{-1} ,T$=$300 K, dt=2\times 10^{-5}$ ms. $N_t=10^4$ trajectories where generated with $N=5000$ samples each.

**Figure 6:**
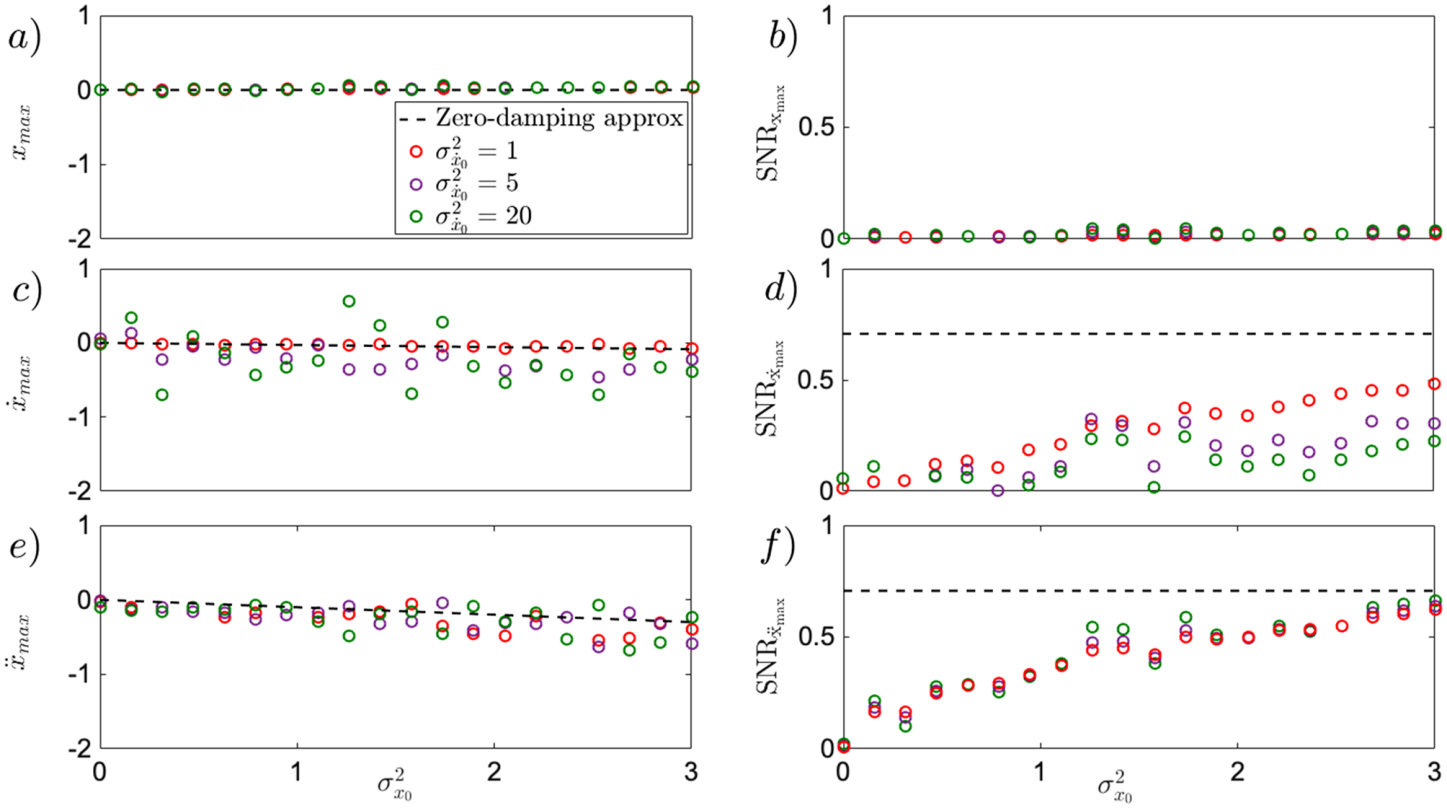
The uncertainty-induced effect in the maxima of distribution for position, instantaneous velocity and acceleration to the uncertainty of the initial velocity state, $\sigma _{{\dot{x}}_0}^2$ **(a,b)**, the evolution of the maximum of position distribution is not modified by increasing initial uncertainty of the velocity state, $\sigma _{{\dot{x}}_0}^2$, similar to its global counterpart $\langle x \rangle$ **(c,d)**, the evolution of maximum of velocity distribution is investigated, showing on **(c)**, almost no sensitivity to small changes in initial velocity uncertainty, but increase requirement of ensemble size. Simultaneously , the $SNR_{{\dot{x}}}$ on **(d)** slowly converges to the $SNR_{{\dot{x}}} =1/\sqrt{2}$ (black dashed line) value but its convergence is reduced with increasing initial velocity uncertainty $\sigma _{{\dot{x}}_0}^2$ **(e,f)**, the local characteristics of instantaneous acceleration, $\ddot{x}_{max}$ **(e)**, displays little to no sensitivity to increasing initial velocity uncertainty, while its $SNR_{\ddot{x}}$ requires a high ensemble size to be simulated ($N_t=10^6$). The convergence of the $SNR_{\ddot{x}}$ **(f)** is not modified by increasing initial velocity uncertainty $\sigma _{{\dot{x}}_0}^2$. The black dashed line correspond to the evolution of maxima of distribution for the zero-damping-limit, shown in Fig.5. Equation (1) has been simulated using $\kappa =6k_BT \upmu \mathrm{m}^{-3} kg^{-1}, T = 300 K,\langle x_0 \rangle =0, \langle {\dot{x}}_0 \rangle =0, t=0.1 ms, dt=2\times 10^{-6}$ ms. $N_t=10^6$ trajectories where generated with $N=5000$ samples each.

To complete the analysis, the dynamics of a levitated particle in cubic potential needs to be characterised also by means of the most-likely position, speed and acceleration. The maxima of the respective distribution are not only a directly measurable characteristic of unstable motion, but they also open a new local use of Maxwell’s deamon in stochastic thermodynamics^27^. Both mean and maximum of instantaneous quantities, stimulated by initial uncertainty, should ideally increase coherently (typical motion in the same direction) with a constant signal-to-noise ratio. Fig.5 shows the dynamics of maximum of position distribution(a,b, red dots), in both high friction regime (a), as well as in low friction regime (b). The latter shifts more pronouncedly, while maintaining its atypical feature observed in^27,30^ for over-damped dynamics. A new feature arises for the-most-likely speed, where both in high and low friction regime, the maximum of average and instantaneous velocity distribution shifts alongside the potential force as seen by the red arrow in Fig. 1 (c,d). A more quantitative dynamics of the latter is shown in Fig.5c showing that the deterministic limit (red line) is reached at damping of $\Gamma =10^{-2}$ Hz. Contrarily to the mean instantaneous velocity, shown in Fig.4, the maximum of velocity distribution does not succumb to increasing initial velocity uncertainty. The curvature on the other hand is responsible for the reduction of the $SNR_{{\dot{x}}_{max}}$, as it becomes larger with increasing initial velocity uncertainty $\sigma _{{\dot{x}}_0}^2$, slowing down the convergence of the $SNR_{{\dot{x}}_{max}}$ as shown in Fig.6d.

The shift of the most-likely acceleration is alongside the potential force, as shown in Fig.1(f, red arrow). It becomes evident only at low pressures, and disappears towards the high friction limit. The sharp shape of the acceleration distribution, displayed in the inset of Fig.5d shows a large shift of mean of instantaneous acceleration, but a small shift of its maximum, although larger compared to the shift of the maximum of velocity distribution. Despite the negligible role that the uncertainty of the initial velocity state displays in the evolution of the maximum of acceleration distribution, as shown in Fig.6e,f, it is of notice the convergence of the $SNR_{\ddot{x}}$ to the $1/\sqrt{2}$, denoted by the dashed black line in Fig.6f.

The minimal requirement to observe a shift of the maximum in the acceleration distribution, is a non-zero uncertainty in the initial velocity state, which mediates from the sharp tail on the right of the distribution, making its maximum hard to identify. Moreover we report that the most-likely acceleration, when the dynamics starts at different initial position $\langle x_0 \rangle \ne 0$, shifts atypically for small values of initial position uncertainty. This is due by a pure deterministic drift in a regime dominated by inertia when the trajectory explore region away from the plateau. Moreover, their distribution shows no visible light tails, contrary to the position distribution in the over-damped regime^27^, where the fast and unstable dynamics of *x* drags all the trajectories quickly to the divergence.

## Conclusion for experimental tests

We have demonstrated the uncertainty-induced instantaneous speed and acceleration of a levitated particle in the highly unstable cubic potential. Moreover, all the simulations have been performed using parameters of current underdamped experiments^12^, together with the parameters of cubic optical potential^29,30^ directly motivating our predictions to be experimentally tested to witness these new nonlinear mechanical phenomena. However, the analysis of the presented regime is also applicable to other underdamped experiments^31–33^. The first point worth experimentally verifying, shown in Figs. 1 and 2, is how the uncertainty-induced effect turns to instantaneous velocity and acceleration, reaching the zero-damping approximation described in Eqs. (5) to  (7) at pressures of $p=10^{-5}$ mbar, and room temperature. Then, the experiment can proceed to investigate how robust the previous result was when the particle, instead of being initially steady at the plateau, possessed nonzero initial velocity $\langle {\dot{x}}_0 \rangle$ as showed in Fig. 3. The latter unveiled an interesting feature, observable in the cubic potential, consisting of stochastic breaking and speeding induced by initial position uncertainty $\sigma _{x_0}^2$. Moreover, remarkably the uncertainty-induced effect can be observed even for slowly moving particles (see blue and green dots in Fig. 3). A parameter that needs to be kept under control is the initial velocity uncertainty, which although can drive the shift of instantaneous acceleration (Fig. 4 orange dots), is disruptive for the measurement of instantaneous velocity (purple and orange dots Fig. 4). Last, but not least, we showed that the maxima of the instantaneous speed, and acceleration distributions shift normally, alongside their respective mean in contrast to the position maximum which maintains its atypical shift as previously observed in the over-damped regime^27,30^ (Fig. 5). The sensitivity of maxima towards increasing initial velocity uncertainty, as shown in Fig. 6, displays a worsening of the accuracy which in turns demands increasing ensemble size. We report that imperfections to the cubic potential, i.e., $V(x) = k_2 x^2 + k_3 x^3$, do not mask the uncertainty-induced effect for small initial uncertainty, provided that $k_2$ is small enough to not dominate the dynamics at transient times around the origin. For single well Gaussian potential in experiment^12^ however, the uncertainty-induced effect is not visible, contrarily to a tilted double well potential (as in^34^) where the uncertainty-induced effect with increasing $\mathrm {SNR}$ can be observed. It enlarges the possibility of upcoming experimental tests with diverse nonlinear potential landscapes.

All these experimental tests will verify new underdamped transient effects of a particle living at the edge of instability, paving the way to explore highly nonlinear stochastic phenomena. Future targets comprise of quantum mechanical analysis of deeply underdamped and highly cooled particles in the unstable cubic potential, initially close to the mechanical ground state.

## Methods

### Numerical simulation with experimental numbers

To numerically simulate the Langevin dynamics described in Eq. (1) using real experimental values, one has to re-scale the equation of motion. Usually, for linear dynamics, the equation is re-scaled using the mechanical Q-factor. With nonlinear systems this is not valid anymore, and a different route must be followed. In the case of Eq. (1), it is useful to re-scale position and time as follows

17 $$\begin{aligned} q = \frac{x}{l}, \qquad {\bar{t}}&= \frac{t}{\tau }, \end{aligned}$$

with *q* being the dimensionless position, *l* the position re-scaler, ${\bar{t}}$ the dimensionless time, and $\tau$ the time re-scaler. It naturally follows from Eq.  (17) that ${\dot{q}}=\tau {\dot{x}}/l$, and $\ddot{q}=\tau ^2 \ddot{x}/l$. In order to determine the values of the re-scaler, one can use, for the position ${\bar{l}}=5\times 10^{-6}$ which is the length of the potential used in experiment^30^, and then focus attention on the positive part of the potential, of length ${\bar{l}}/2$. Subsequently choose a point, *P* belonging to it, with coordinate (*xp*, *yp*). With this point one can build a straight line passing through the origin and the chosen point, and notice that it forms a triangle containing the piece of potential up to point *P* chosen. The angle between the origin and the hypotenuse is then given by the stiffness *k* of the potential. Subsequently, the position re-scaler can be calculated as $l=2\sqrt{A/k}$, where *A* denotes the area of the triangle previously built, and the factor 2 comes from the fact that we extend the calculation to the whole domain of the cubic potential. The re-scaler *l* calculated, now has a clear dependence on the stiffness of the potential $K\approx k_BT \mu m^{-3}$, and subsequently on the chosen temperature *T*, and it varies between $10^{-3}\mu m< l < 10^{-2}\mu m$. The time re-scaler, $\tau$ is calculated by numerically computing the first passage time for the selected parameters.

Following the re-scaling procedure explained before, the Langevin equation can be transformed into

18 $$\begin{aligned} \ddot{q} = -\gamma \tau {\dot{q}} - {\bar{k}}\tau ^2 l q^2 + \frac{\tau ^2}{l}\sqrt{\frac{2D}{\tau }}{\bar{\xi }}(t), \end{aligned}$$

where ${\bar{k}}=\kappa /l^3$, and the mass *m* has been absorbed by $\kappa =K/m$, $\gamma =\Gamma /m$, $D=k_BT\gamma /m$, and ${\bar{\xi }}(t) = \sqrt{\tau ^{-1}} \xi (t)$. The term ${\bar{k}}=\kappa /l^3$ comes from the re-normalisation of the potential stiffness, done to render the dimensions of the new stiffness, ${\bar{k}}=[1/(m s^2)]$, such that the potential term, ${\bar{k}}\tau ^2 l q^2$ becomes a-dimensional in the re-scaling.

### Uncertainty-induced effects for non vanishing initial position

**Figure 7:**
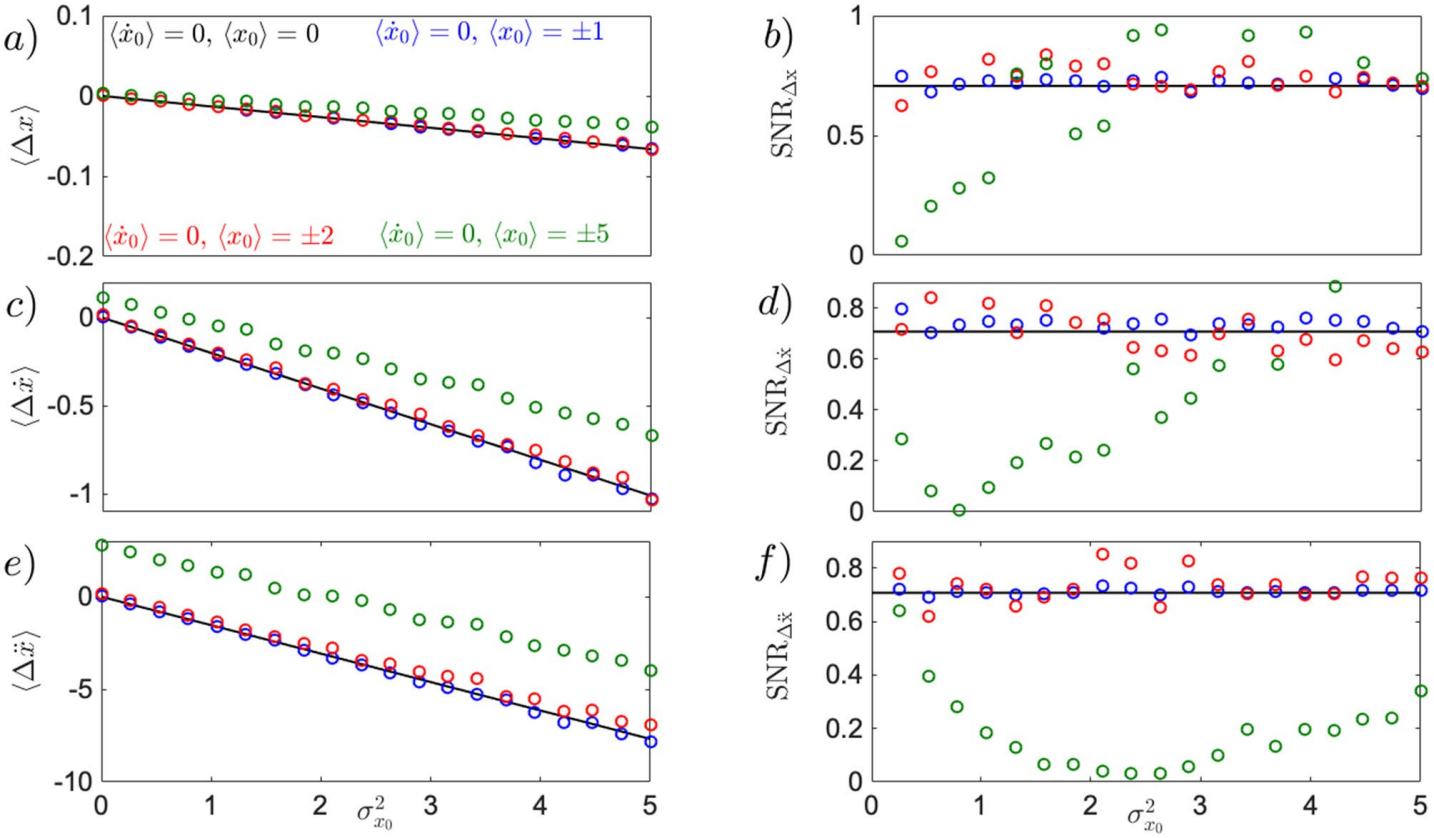
Uncertainty-induced effects for non vanishing initial position $\langle x_0 \rangle$ for position statistics **(a,b)**, instantaneous velocity statistics **(c,d)**, and instantaneous acceleration statistics **(e,f)**. All simulations (dots) have been performed based on Eq. (1) using $\sigma _{{\dot{x}}_0}^2=0$, and $\langle {\dot{x}}_0 \rangle =0$. Positioning the particle in different parts of the plateau region (blue dots), reveals no introduction of higher order term of the time series for neither position, velocity, nor acceleration. Their dynamics is well described by Eqs. (14)–(16). Similarly, by positioning the particle away from the plateau, where the nonlinearity starts to become relevant (red dots), Eqs. (14)–(16) still hold to describe dynamics of position, velocity, and acceleration. A different case is when the particle is positioned in a highly nonlinear region of the potential (green dots), i.e., down the slope of the cubic potential (negative values), or on the cubic wall (positive values). Such positions highly affect the dynamics, introducing new terms of the time series, where Eqs. (14)–(16) are no longer valid. As a result, the position still displays the uncertainty-induced feature, witnessed by the increasing $SNR_{\Delta x}$ **(b)** that converges to the constant value $1/\sqrt{2}$. Velocity **(d)** and acceleration **(f)** display a *SNR* initially decreasing to zero, after which the uncertainty-induced feature becomes visible once again, converging with different speed of growth to the constant value $1/\sqrt{2}$ for larger values of initial position uncertainty. Equation (1) has been simulated using $\kappa =6k_BT \upmu \mathbf{m}^{-3} \mathbf{kg}^{-1},T = 300 K,\sigma _{{\dot{x}}_0}^2 =0, \langle {\dot{x}}_0 \rangle =0, dt=2\times 10^{-5}$ ms. $10^4$ trajectories where generated with 5000 samples each.

Throughout the paper we have always assumed to have a particle initially positioned at the inflection point $\langle x_0 \rangle =0$. It becomes natural to ask how different $\langle x_0 \rangle$ affect the uncertainty-induced effect described. As evidenced in Fig. 7, for small nonzero $\langle x_0 \rangle =1$ (blue dots), Eqs. (14)–(16) still describe the dynamics for position (a,b), velocity (c,d), and acceleration (e,f), where their *SNR* (b,d,f) show no deviation from $1/\sqrt{2}$. A different case is the large non zero $\langle x_0 \rangle$ (green dots) for which Eqs. (14)–(16) no longer holds. In this case, the particle already feels strong nonlinear terms, which require more terms in the time expansion from  (4). While the respective *SNR* undergo an initial decrease (for velocity and acceleration), they all converge to $1/\sqrt{2}$ for increasing initial position uncertainty $\sigma _{x_0}^2$. To preserve the short time dynamics at such nonlinear positions, a decrease of the time scale seems necessary (a tenth of $t=0.1$ms is sufficient for the parameters used in Fig. 7 to produce the green dots).
